# Veal Calves Produce Less Antibodies against *C. Perfringens* Alpha Toxin Compared to Beef Calves

**DOI:** 10.3390/toxins7072586

**Published:** 2015-07-10

**Authors:** Bonnie R. Valgaeren, Bart Pardon, Evy Goossens, Stefanie Verherstraeten, Sophie Roelandt, Leen Timbermont, Nicky Van Der Vekens, Sabrina Stuyvaert, Linde Gille, Laura Van Driessche, Freddy Haesebrouck, Richard Ducatelle, Filip Van Immerseel, Piet Deprez

**Affiliations:** 1 Department of Large Animal Internal Medicine, Faculty of Veterinary Medicine, Ghent University, Salisburylaan 133, 9820 Merelbeke, Belgium; E-Mails: bart.pardon@ugent.be (B.P.); nicky.vandervekens@ugent.be (N.V.D.V.); sabrina.stuyvaert@ugent.be (S.S.); linde.gille@ugent.be (L.G.); lauvdrie.vandriessche@ugent.be (L.V.D.); piet.deprez@ugent.be (P.D.); 2 Department of Pathology, Bacteriology and Avian Diseases, Faculty of Veterinary Medicine, Ghent University, Salisburylaan 133, 9820 Merelbeke, Belgium; E-Mails: evy.goossens@ugent.be (E.G.); stefanie.verherstraeten@ugent.be (S.V.); Leen.Timbermont@ugent.be (L.T.); Freddy.Haesebrouck@ugent.be (F.H.); Richard.Ducatelle@ugent.be (R.D.); Filip.vanimmerseel@ugent.be (F.V.I.); 3 Unit for Coordination of Veterinary Diagnosis, Epidemiology and Risk Assessment (CVD-ERA), Veterinary and Agrochemical Research Centre (VAR-CODA-CERVA), Groeselenberg 99, 1180 Uccle, Brussels, Belgium; E-Mail: sophie.roelandt@coda-cerva.be

**Keywords:** alpha toxin, antibodies, *Clostridium perfringens*, enterotoxaemia, perfringolysin, veal

## Abstract

Enterotoxaemia is a disease with a high associated mortality rate, affecting beef and veal calves worldwide, caused by *C. perfringens* alpha toxin and perfringolysin. A longitudinal study was conducted to determine the dynamics of antibodies against these toxins in 528 calves on 4 beef and 15 veal farms. The second study aimed to determine the effect of solid feed intake on the production of antibodies against alpha toxin and perfringolysin. The control group only received milk replacer, whereas in the test group solid feed was provided. Maternal antibodies for alpha toxin were present in 45% of the veal calves and 66% of the beef calves. In beef calves a fluent transition from maternal to active immunity was observed for alpha toxin, whereas almost no veal calves developed active immunity. Perfringolysin antibodies significantly declined both in veal and beef calves. In the second study all calves were seropositive for alpha toxin throughout the experiment and solid feed intake did not alter the dynamics of alpha and perfringolysin antibodies. In conclusion, the present study showed that veal calves on a traditional milk replacer diet had significantly lower alpha toxin antibodies compared to beef calves in the risk period for enterotoxaemia, whereas no differences were noticed for perfringolysin.

## 1. Introduction

Enterotoxaemia is a fatal disease of young cattle, particularly in intensive production systems, and may be characterized by sudden death and necro-haemorrhagic enteritis [1]. The pathogenesis of enterotoxaemia in calves has long been unclear. Recently, *Clostridium perfringens* alpha toxin and perfringolysin have been identified as the key virulence factors involved in the development of bovine necro-haemorrhagic enteritis [2].

Belgian Blue (BB) calves are predisposed to enterotoxaemia. In BB veal calves up to 20% of the total mortality, especially in the last weeks before slaughter, can be attributed to enterotoxaemia, whereas in Holstein Friesian (HF) or crossbred veal calves the incidence is significantly lower [3,4]. High mortality rates due to enterotoxaemia have also been reported in suckler calves, whereas enterotoxaemia is less frequent in beef production systems with immediate separation from the dam [5,6,7]. In addition to a possible genetic predisposition, the differences in diet between veal and beef calves might play a role [8]. Whereas veal calves are mainly raised on milk replacer and receive only limited amounts of solid feed, beef calves are fed a limited amount of milk replacer, are weaned at an early age, and thereafter predominantly fed with solid feeds [9,10]. Similar to other animal species, the rich diet of veal calves has been implicated in predisposition to enterotoxaemia, but there is no scientific evidence for this statement [11,12,13].

In other clostridial diseases, maternal immunity against exotoxines is generally protective [14]. In calves, experimental studies have shown maternal antibodies against *C. perfringens* epsilon and alpha toxin are detectable up to 200 days after birth [15,16]. To date no information on maternal antibody decline and acquisition of active immunity against alpha toxin and perfringolysin in calves is available. Such information may help to elucidate the epidemiology of bovine enterotoxaemia and is of crucial importance for the development of alpha toxin and perfringolysin vaccination schemes in the field. We hypothesized that these antibody dynamics differ between breeds and production systems, thereby partially explaining differences in susceptibility for enterotoxaemia.

Therefore the first objective of the present study was to determine antibody dynamics in beef calves and veal calves of different breeds. The second objective was to determine the effect of solid feed intake on the production of alpha toxin and perfringolysin antibodies in veal calves.

## 2. Results and Discussion

### 2.1. Study 1: Antibody Dynamics against Alpha Toxin and Perfringolysin in Veal and Beef Calves

#### 2.1.1. Alpha Toxin Antibody Dynamics

**Figure 1:**
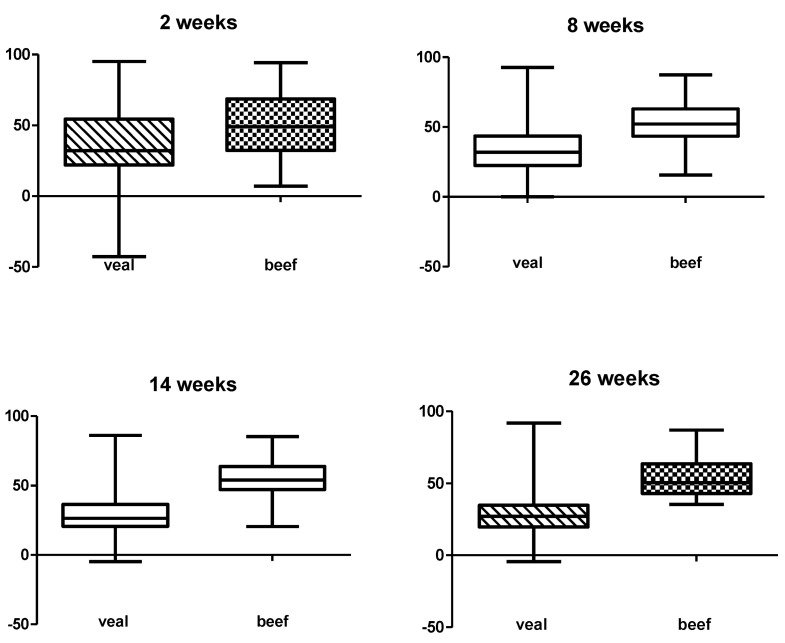
Results of the *Clostridium perfringens* alpha toxin antibody blocking ELISA in study 1, presented as the mean percentage inhibition of the OD for veal and beef calves at every sampled age.

Figure 1 and Table 1 give an overview of the mean inhibition of the optical density (OD) and Table 1 of the prevalence of *C. perfringens* alpha toxin antibodies in the different groups at the different ages. At the age of two weeks, the seroprevalence of alpha toxin was 45% and 66% in veal and conventional BB respectively. This difference was not significant. In beef calves, a smooth transition from maternal to actively produced alpha toxin antibodies was noted with no significant differences between the different ages. In contrast, veal calves demonstrated a significant decline in alpha toxin antibodies between the ages of two and eight weeks as well as between the ages of 8 and 14 weeks. Only 5% ± 4% of the veal calves seroconverted for alpha toxin over the course of this study. The difference between subjects (production system beef *vs.* veal) was significant at every time point (*p* < 0.001). At the age of 26 weeks, the percentage of animals with alpha toxin antibodies was significantly higher in beef (85%) than in veal calves (16%).

Within the group of veal calves, there was no overall significant effect of breed on % inhibition of OD. At the age of 8 and 14 weeks crossbred calves had significantly higher % inhibition of OD (more antibodies) than HF calves (*p* < 0.01).

**Table 1:** Results of the *C. perfringens* alpha toxin antibody blocking ELISA.

Alpha toxin blocking ELISA		Mean % Inhibition of OD ± Standard Deviation				Seroprevalence ± Standard Deviation			
		Age (in weeks)				Age (in weeks)			
Populations	*n*	2	8	14	26	2	8	14	26
Veal total	443	^a^43 ± 21	^b^35 ± 17	^c^30 ± 15	^c^30 ± 15	46 ± 13	29 ± 14	17 ± 13	16 ± 9
Veal cross	167	^a^45 ± 22	^b^40 ± 18	^c^35 ± 15	^c^33 ± 15	49 ± 18	45 ± 11	28 ± 12	20 ± 12
Veal HF	121	^a^40 ± 21	^b^29 ± 12	^b^23 ± 10	^b^29 ± 17	43 ± 10	17 ± 4	6 ± 10	17 ± 8
Veal BB	155	^a^42 * ± 20	^b^34 * ± 17	^c^29 * ± 24	^c^26 * ± 13	45 ± 13	26 ± 10	16 ± 4	9 ± 6
Beef BB	85	^a^51 * ± 23	^a^52 * ± 16	^a^55 * ± 13	^a^53 * ± 12	66 ± 40	76 ± 28	91 ± 8	85 ± 14
All calves	528	^a^44 ± 21	^b^37 ± 18	^b^32 ± 16	^b^33 ± 17	44 ± 22	37 ± 18	32 ± 16	33 ± 17

BB: Belgian Blue; HF: Holstein Friesian; cross: crossbred; Remarks: Statistic analyses were performed on the Mean % inhibition of OD. Values indicated with * are significantly different (*p* < 0.05) between veal and beef Belgian Blue calves at a given time point. Within subjects effect was significant in the veal groups. Values with a different letter (a,b,c) are significantly different (*p* < 0.05).

#### 2.1.2. Perfringolysin Antibody Dynamics

**Figure 2:**
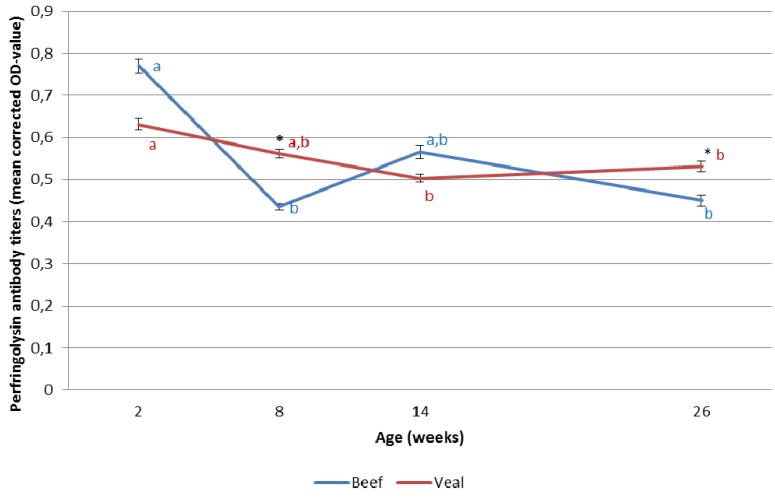
*Clostridium perfringens* perfringolysin antibody dynamics in 153 Belgian blue veal and 84 beef calves. Values indicated with * are significantly different (*p* < 0.05) between veal and beef at a given time point. Within subjects effect was significant in the veal group. Values with a different letter are significantly different within the test group (veal or beef) (*p* < 0.05). Error bars represent standard error of the mean.

Results are summarized in Figure 2. Perfringolysin antibodies decreased significantly between two and eight weeks of age (*p* < 0.01) in veal calves and between 2 and 14 weeks of age in beef calves. Veal calves had significantly higher perfringolysin antibody titers than beef calves at the age of eight weeks and at the age of 26 weeks (*p* < 0.01).

### 2.2. Study 2: Effect of Solid Feed Provision on Alpha Toxin and Perfringolysin Antibody Dynamics

#### 2.2.1. Mortality, Incidence of Enterotoxaemia and Average Weight Gain

There was no morbidity during the trial, so no calves had to be excluded from the experiments; nor were there any fatalities or enterotoxaemia cases.

Average body weight at two weeks of age was 53 ± 6 kg in group 1 and 52 ± 3 kg in group 2. Average daily gain was significantly higher in the calves that received solid feed (836 ± 118 g *vs.* 1002 ± 120 g; *p* < 0.05, two way ANOVA).

#### 2.2.2. Alpha Toxin and Perfringolysin Antibody Dynamics

**Figure 3:**
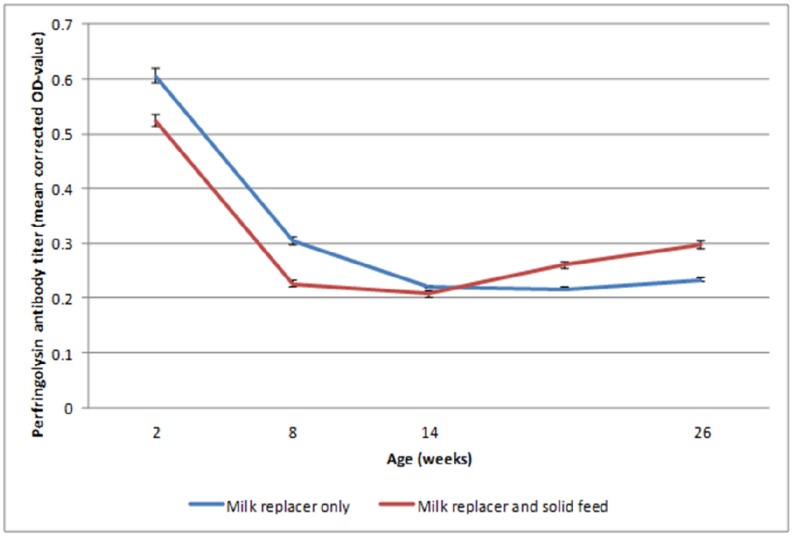
Dynamics of *Clostridium perfringens*’ perfringolysin antibody titers for Holstein Friesian veal calves fed only with milk replacer or with milk replacer and solid feed. Error bars represent standard error of the mean.

There were no significant differences in alpha toxin or perfringolysin antibody levels between the groups (Table 2 and Figure 3). For alpha toxin, antibodies were high in both groups during the whole trial and the overall seroprevalence was 100%. For perfringolysin, there was a significant decrease in antibody titers between two weeks and eight weeks of age in both groups (*p* < 0.05).

**Table 2:** Results of study 2: effect of solid feed provision.

Alpha Toxin Serology (% Inhibition of OD ± Standard Deviation)				
Group	Age in Weeks			
	2	8	14	26
Group 1	72 ± 14	67 ± 11	67 ± 8	70 ± 5
Group 2	64 ± 13	66 ± 8	68 ± 6	67 ± 5
Total	68 ± 14	66 ± 10	68 ± 7	69 ± 5

All calves were Holstein Friesian male veal calves. Group 1 (*n* = 15) was fed with only milk replacer, while group 2 (*n* = 15) received milk replacer and solid feed. There were no significant differences between groups or within a group between time points.

### 2.3. Discussion

To be able to protect calves from enterotoxaemia by vaccination, it is crucial to obtain insights in the presence and decline of maternal antibodies against the toxins included in the vaccine [14]. In this study, maternal antibodies against alpha toxin were common in calves destined for beef and veal production, as has also been described for epsilon toxin in goats [14]. In veal calves, there was a significant decrease in antibodies against alpha toxin until the age of 14 weeks and the antibody levels remained low until the end of the study when the calves were 26 weeks of age. At the age of 14 weeks, only 17% of the veal calves were seropositive for alpha toxin, possibly indicating that, from that moment on, the majority of these animals could have been vaccinated with alpha toxin-based vaccines without interference with maternally derived antibodies. Since in veal calves, enterotoxaemia mainly occurs from the age of 20 weeks onward, it might be possible to immunize them prior to the period of greatest risk, using such vaccines. Further studies, including field trials and protection studies in experimentally infected calves are, however, necessary to try and develop effective vaccination schedules.

We hypothesized that differences exist between veal and beef calves in dynamics of alpha toxin and perfringolysin antibodies, which might help to explain the higher incidence of enterotoxaemia in veal calves [4]. This hypothesis was confirmed for alpha toxin. Indeed, only 5% of the veal calves, raised with the traditional all-liquid diet, seroconverted for alpha toxin and at the age of 26 weeks, only 16% of the veal calves were seropositive for alpha toxin, as opposed to 85% of the beef calves. Based on the absence of a breed effect within the same nutritional management (=veal), it was hypothesized that the predisposition of veal calves might be associated with the diet rather than the breed. One possible explanation might be the effect of the diet on the production of alpha toxin by *C. perfringens* in the gastro-intestinal tract. Prolonged production of low amounts of toxin in the gastro-intestinal might be influenced by dietary factors and may lead to seroconversion and possibly protection against enterotoxaemia. This has also been suggested for acquisition of antibodies against tetanus toxin and *C. perfringens* epsilon toxin [14,17,18,19,20,21].

Our hypothesis was not confirmed for perfringolysin antibodies, since at the ages of 8 and 26 weeks, veal calves had significantly higher antibody titers against this toxin than beef calves. It has been shown that alpha toxin and perfringolysin act synergistically in the development of necrohemorrhagic enteritis in a calf intestinal loop model, possibly by targeting the endothelial cells [2]. The relative importance of antibodies against both toxins in protection requires further studies.

Since the most striking difference between the diet in veal and beef calves is the availability of large amounts of solid feed, we studied the influence of solid feed provision. Unfortunately, we encountered several unexpected observations which made the interpretation of this trial more difficult. First, using the same ELISA, all of the studied calves had high levels of alpha toxin antibodies at the age of two weeks, in contrast to the field observations made between 2009 and 2011. A straightforward explanation was not found. It is possible that differences in colostrum provision or in the vaccination status of the dams may have played a role. Secondly, in contrast to the first study, the veal calves on the all-liquid diet produced alpha toxin antibodies. Differences in milk replacer composition may help to explain this observation. At the time of the first study (2009–2011), veal calves were predominantly fed with high amounts of highly concentrated milk replacer. The second study was conducted five years later, and alternative feeding regimes claiming improved animal welfare had then become common practice in the local veal industry. The current milk replacers, including the product used in our second study, contain lower concentrations of whey proteins than milk replacers generally used at the time of our first field study in veal calves. Moreover, the current feeding regimes also provide lower amounts of milk replacer compared to the previous regimes. Contact with milk protein has been observed to decrease alpha toxin production by *C. perfringens*, which may influence antibody production against this toxin ([22], confirmed by unpublished data in supplementary materials). Since the provision of solid feed did not influence the antibody dynamics of *C. perfringens* alpha toxin and perfringolysin, differences in quantity and quality of milk replacer used in veal calves compared to beef calves might lead to different exposure to alpha toxin, possibly explaining the difference in antibody production observed in both studies. Further research is required to unravel the dietary risk factors for enterotoxaemia (quantity, concentration and composition of both milk replacer and solid feed), in order to be able to prevent the disease through nutritional management.

## 3. Experimental Section

All experiments were conducted with permission of the local ethical committee (EC2012/118 and EC2014/016).

### 3.1. Study 1: Dynamics of Antibodies against Alpha Toxin and Perfringolysin in Veal and Beef Calves

A prospective longitudinal cohort study was conducted to determine the effect of production systems (veal or beef) on the alpha toxin and perfringolysin antibody dynamics and of breed on the alpha toxin antibody dynamics in veal calves.

#### 3.1.1. Animals

The study was conducted in 528 animals, housed on 19 conveniently selected farms (15 veal farms, 5 BB, 5 Holstein Friesian (HF), 5 crossbred (HFxBB)) and four beef farms (4 BB) (Table 3). Veal calves were sampled between October 2007 and October 2009, and beef calves were sampled between November 2013 and August 2014. Veal calves received a commercial all liquid diet, with minimal amounts of solid feed (<200 g/day). On the conventional BB farms calves were weaned between 14 and 26 weeks of age. Details on the rations and housing conditions in veal and beef farms are provided in Table 3. None of the farms had a history of vaccination against enterotoxaemia. On the beef farms, calves received colostrum from unvaccinated dams. For the veal farms, vaccination history of the dams was unknown.

Beef calves were weaned (deprived of milk replacer) at the age of about 12 weeks, whereas veal calves received the milk replacer throughout the study.

**Table 3:** Housing conditions and feeding regime of the herds included in the first study on antibody dynamics of *C. perfringens* alpha toxin and perfringolysin.

Herd	Number of Calves in Study	Production System	Breed	Floor	Feed			
					Milk Replacer			
					% NP	% SMP	Weaning	Solid Feed
1	25	Veal	HF	Slatted floor	95	5	No	<200 g/d
2	25	Veal	HF	Slatted floor	95	5	No	<200 g/d
3	25	Veal	HFXBB	Slatted floor	95	5	No	<200 g/d
4	25	Veal	BB	Slatted floor	30	70	No	<200 g/d
5	25	Veal	HFXBB	Slatted floor	95	5	No	<200 g/d
6	25	Veal	BB	Slatted floor	30	70	No	<200 g/d
7	25	Veal	HF	Slatted floor	95	5	No	<200 g/d
8	26	Veal	HF	Slatted floor	95	5	No	<200 g/d
9	25	Veal	HF	Slatted floor	95	5	No	<200 g/d
10	24	Veal	BB	Slatted floor	30	70	No	<200 g/d
11	30	Veal	BB	Slatted floor	30	70	No	<200 g/d
12	39	Veal	HFXBB	Slatted floor	95	5	No	<200 g/d
13	38	Veal	BB	Slatted floor	30	70	No	<200 g/d
14	41	Veal	HFXBB	Slatted floor	95	5	No	<200 g/d
15	39	Veal	HFXBB	Slatted floor	95	5	No	<200 g/d
16	21	Beef	BB	Straw bedded	0	100	Yes	Ad lib
17	20	Beef	BB	Straw bedded	0	100	Yes	Ad lib
18	27	Beef	BB	Straw bedded	0	100	Yes	Ad lib
19	23	Beef	BB	Straw bedded	0	100	Yes	Ad lib

BB: Belgian Blue; HF: Holstein Friesian; HFXBB: Crossbred calves; SMP: Milk replacer containing whey powder and skimmed milk powder; NP: Nill product (Milk replacer based on whey powder, without skimmed milk powder, casein fraction replaced by vegetable protein); Ad lib: unrestrained provision.

#### 3.1.2. Sampling

Serum samples were taken from the jugular vein from all calves at the age of 2, 8, 14 and 26 weeks. All serum samples were kept frozen (−18 °C) until analysis.

#### 3.1.3. Antibody Determination

##### Alpha Toxin Antibody ELISA

A commercial serum blocking Elisa kit for *C. perfringens* alpha toxin (BIO K 291, Bio-X, Jemelle, Belgium) was used according to the manufacturer’s guidelines. The percentage inhibition for a sample was calculated by the following formula:

(1) % inhibition sample = [(OD neg – OD sample)/OD neg] × 100

A calf was considered seropositive if the inhibition percentage was higher than 40%. A calf was considered to have seroconverted if the inhibition persentage increased with 40% of more in consecutive sampling points.

##### Expression and Purification of Perfringolysin

The pTrcHisA plasmids encoding native perfringolysin (Department of Microbiology and Immunology, College of Medicine, University of Oklahoma, USA) were transformed in chemically competent *E. coli* TunerTM(DE3)pLysS cells (Novagen, Darmstadt, Germany). The proteins were expressed in Terrific Broth (24% Tryptone (Oxoid, Hampshire, UK), 42% Yeast extract (BD), 4% glycerol, 0.72 M Na_2_HPO_4_, 0.16 M NaH_2_PO_4_) and the protein expression was induced with 0.5 mM isopropyl β-d-thiogalactopyranoside (IPTG, Promega, Madison, WI, USA) [22]. The *E. coli* cells were lysed by sonication in a lysis buffer (pH 7.4) containing 20 mM NaPO_4_, 0.5 M NaCl, 20 mM Imidazole, 1 mg/mL lysozyme (Sigma-Aldrich, St. Louis, MO, USA) supplemented with protease-inhibitor cocktail (Sigma-Aldrich, St. Louis, MO, USA). The supernatant was then loaded on a Cobalt-affinity column (His GraviTrap; GE Healthcare, Brussels, Belgium). The column was washed with buffer (20 mM NaPO_4_, 0.5 M NaCl) containing 40 mM Imidazole and the histidine-tagged proteins were eluted from the column with buffer containing 0.3 M imidazole. The eluted proteins were dialyzed against phosphate-buffered saline (PBS—137 mM NaCl, 2.7 mM KCl, 10 mM Na_2_HPO_4_, 1.8 mM KH_2_PO_4_; pH 7.4) overnight at 4 °C. Protein concentrations were determined with the Pierce BCA protein Assay (Thermo Scientific, Waltham, MA, USA) using bovine serum albumin (Sigma-Aldrich, St. Louis, MO, USA) as a standard. Protein purity was analyzed by sodium dodecyl sulfate-polyacrylamide gel electrophoresis (SDS-PAGE) with Coomassie Brilliant Blue Staining (Sigma-Aldrich, St. Louis, MO, USA). PageRuler Unstained Protein Ladder (Thermo Scientific, Waltham, MA, USA) was used as a protein standard.

##### Perfringolysin Antibody ELISA

A direct ELISA was developed to detect antibodies against perfringolysin in serum samples. Immunoplates (Nunc-Immunoplate Polysorp) were coated with 20 μg/mL of purified perfringolysin diluted in carbonate buffer (pH 9.5) and incubated overnight at 4 °C. After washing the plates with phosphate-buffered saline (PBS) containing 0.1% Tween 20 (Washing Buffer, WB), test and control sera diluted in PBS containing 0.05% Tween 20 (Dilution Buffer, DB) were added to the plates and incubated for 1 h at 37 °C. The plates were washed between steps with WB, pH 7.4. A hyperimmune serum from vaccinated calves diluted in 1:100,000 DB was used as positive control. Testsera and negative control serum from a neonatal calf deprived of colostrum were diluted 1:50. All serum dilutions were performed in duplicate. The plates were incubated for 2 h at 37 °C with the sera and subsequently incubated for 1.5 h at 37 °C conjugated with α bovine horseradish peroxidase (HRP), diluted 1:30,000, and again incubated for 1.5 h at 37 °C. The reaction was developed with 3,3′,5,5′-Tetramethylbenzidine (TMB; Sigma–Aldrich, St. Louis, MO, USA) as chromogen substrate and the intensity of staining was read at 450 nm after 30 min of incubation. All reagents were purchased from Sigma–Aldrich, St. Louis, MO, USA. The perfringolysin antibody ELISA was only done on samples from BB beef and veal.

#### 3.1.4. Statistics

To determine the effect of breed and production system (beef *vs.* veal) on alpha toxin ELISA inhibition percentage and perfringolysin ELISA optical density, a linear mixed model with repeated measurements was used (PROC MIXED). Sampling point was added as the repeated effect and herd as a random effect to account for clustering of calves within a herd. Test variables were breed (BB, HF, or crossbred) and production system (beef *vs.* veal). Pairwise comparisons of significant main effects were made using Bonferroni corrections. Significance was set at *p* < 0.05. All analyses were done in SAS 9.4 (SAS Institute, Cary, NC, USA).

### 3.2. Study 2: Effect of Solid Feed Intake on Alpha Toxin and Perfringolysin Antibody Dynamics

#### 3.2.1. Study Design

A longitudinal experimental cohort study to determine the effect of solid feed intake on perfringolysin and alpha toxin antibody dynamics in Holstein Friesian veal calves was conducted between May 2014 and November 2014. To detect a difference of 25% in calves that are seropositive against alpha toxin with 95% confidence and 80% power, a sample size of 12 calves per group was necessary. A total of 15 calves per group were sampled to account for possible mortality. Serum samples were taken at the age of 2, 8, 14 and 26 weeks. Calves were weighed at the age of 2 and 26 weeks.

#### 3.2.2. Animals, Housing and Feeding

The trial was performed at a commercial veal fattening unit. Thirty male HF calves were randomly assigned to one of two treatment groups at the day of arrival at two weeks of age. All calves were kept on slatted floor without bedding. Both groups were fed the same amount of milk replacer (21.2% crude protein, 17.7% crude fat at dry mater base; Nillproduct, Vilatca nv, Geel, Belgium) in the same concentration, twice a day. At the age of two weeks the calves were fed 220 g MR/feeding, increasing to 1260 g/feeding at the age of 26 weeks. Group 2 also received increasing amounts (32 to 710 g/feeding) of commercial veal solid feed (a mixture of barley, corn, spelt and 10% straw; ash 2.4%, crude protein 9.6%, crude fat 2.9%, crude fiber 8%) twice daily. The vaccination status of the dams was unknown.

#### 3.2.3. Laboratory Analysis

Alpha toxin and perfringolysin antibody ELISAs were performed on all samples as described above.

#### 3.2.4. Statistics

The effect of the treatment group (solid feed or not) on alpha toxin ELISA % inhibition and perfringolysin antibody ELISA OD was determined by a repeated measurements linear mixed model (PROC MIXED) as described above, but without a random effect.

## 4. Conclusions

This study explored antibody dynamics for alpha toxin and perfringolysin in beef and veal calves. A significant difference in the antibody production for alpha toxin, but not for perfringolysin was observed between veal and beef calves. In beef calves a fluent transition from maternal to active immunity for alpha toxin was observed, whereas in veal calves on a traditional all-liquid diet a significant decline of alpha toxin antibodies was noted. Breed did not influence the antibody dynamics. These observations indicate that dynamics of alpha toxin antibodies may influence the occurrence of enterotoxaemia in calves.

## Supplementary Materials

Supplementary materials can be accessed at: http://www.mdpi.com/2072-6651/7/7/2586/s1.
