# Supplementary material for: Veal Calves Produce Less Antibodies against C. Perfringens Alpha Toxin Compared to Beef Calves
Source: Toxins (Basel). 2015 Jul 10;7(7):2586–97. doi: 10.3390/toxins7072586 (PMC4516930; doi:10.3390/toxins7072586)
Supplement: Supplementary file 1 [file toxins-07-02586-s001.pdf]

Supplementary Materials

Milk Lowers the Alpha Toxin Production by *Clostridium Perfringens*

1. Introduction

Enterotoxaemia is a multifactorial disease in young cattle, caused by *Clostridium perfringens* alpha toxin and perfringolysin O. In an intestinal loop model, enterotoxaemia can be induced by injecting *C. perfringens* cultures together with milk replacer (Valgaeren *et al.*, 2013 [1]). This study determines the effect of milk replacer on the toxin production of *C. perfringens*.

2. Methods

Bacterial Strain and Growth Conditions

The *C. perfringens* strains used in this study originated from a case of bovine necro-hemorrhagic enteritis (BCP62) [1]. The strains were grown anaerobically at 37 °C in TGY broth (3% tryptone, 2% yeast extract, 0.1% glucose, 0.1% L-cysteine HCl) supplemented with different concentrations of commercial milk replacer (Vitaspray, Nuscience Drongen, Belgium) sterilized using gamma irradiation (cobalt-60, 25.5 kGy). Growth was monitored by plating ten-fold dilutions of the given time-points on Columbia agar (Oxoid, Basingstoke, UK) supplemented with 5% defibrinated sheep blood. Cell-free supernatants from the *C. perfringens* cultures were obtained by centrifugation followed by filtration of the supernatants through a 0.22 µm filter.

3. Toxin Assays

To determine the alpha toxin activity in the supernatants, the lecithinase acitivity was assayed in an egg yolk agar diffusion assay. Therefore 10 µl of the tested supernatants was spotted on Columbia agar (Oxoid, Hampshire, UK) supplemented with 2% (vol/vol) egg yolk. Plates were incubated at 37 °C for 24 h and scanned with a GS-800 calibrated densitometer (Bio Rad Laboratories, Hercules, CA, USA). The opacity of the spots was measured using Quantity One software (Bio Rad Laboratories).

Both assays were performed in duplicate.

4. Results

The effect of milk replacer on the alpha toxin production was monitored by detecting the lecithinase activity of *C. perfringens* grown in the presence of different concentrations of milk replacer. When *C. perfringens* was grown in medium supplemented with a high concentration of milk replacer (10%), the supernatant contained approximately 5 times less alpha toxin than when *C. perfringens* was grown without milk replacer (0%) (Figure S1b). Supplementation of the culture medium with lower amounts of milk replacer, resulted in higher alpha toxin production, compared to the culture grown in the presence of 10% milk replacer.

Supplementation of *C. perfringens* growth medium with different concentrations of milk replacer has no effect on the growth rate of *C. perfringens* (Figure S1a).

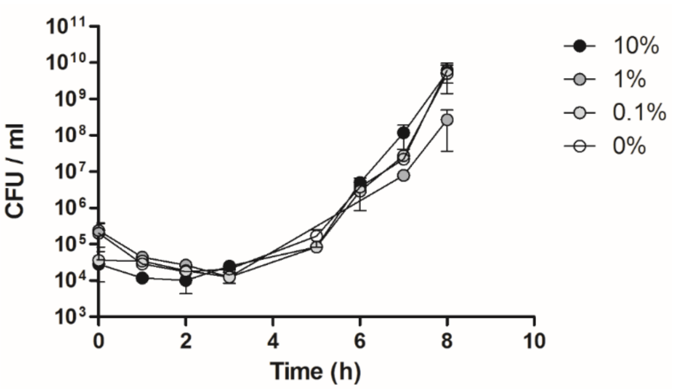

(**a**)

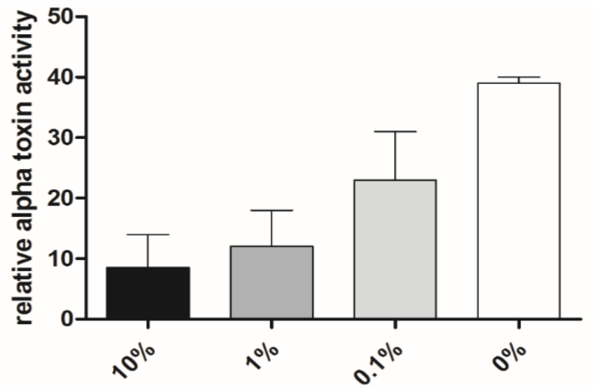

(**b**)

Figure S1. Effect of milk replacer on the growth and alpha toxin production of *C. perfringens.* (**a**) Growth rate of *C. perfringens* in TGY medium alone (0%, white) or supplemented with 10% (black), 1% (dark grey) or 0.1% (light grey) milk replacer. The dots represent the mean with the standard error of the means; (**b**) The alpha toxin production of *C. perfringens* grown in TGY medium alone (0%, white) or supplemented with 10% (black) 1% (dark grey) or 0.1% (light grey) milk replacer was determined by measuring the lecithinase activity in an egg yolk agar assay. The bars represent the mean with the standard error of the means.

5. Conclusions

The addition of milk replacer to the culture medium decreases the alpha toxin production of *C. perfringens*. This effect cannot be attributed to an influence of the milk proteins on the bacterial growth, as increasing amounts of milk replacer in the culture medium had no effect on the growth of *C. perfringens.*

Reference

1. Valgaeren, B.; Pardon, B.; Goossens, E.; Verherstraeten, S.; Schauvliege, S.; Timbermont, L.; Ducatelle, R.; Deprez, P.; Van Immerseel, F. Lesion Development in a New Intestinal Loop Model Indicates the Involvement of a Shared *Clostridium perfringens* Virulence Factor in Haemorrhagic Enteritis in Calves. *J. Comp. Pathol.* **2013**, *149*, 103–112.
